# A Preliminary Investigation on Plasma Cell Adhesion Molecules Levels by Protein Microarray Technology in Major Depressive Disorder

**DOI:** 10.3389/fpsyt.2021.627469

**Published:** 2021-04-12

**Authors:** Wanying Liu, Yanqun Zheng, Fuxu Zhang, Mo Zhu, Qian Guo, Hua Xu, Caiping Liu, Haiying Chen, Xiaoliang Wang, Yao Hu, Tianhong Zhang, Zhiguang Lin, Chen Zhang, Guanjun Li, Kaida Jiang, Xiaohua Liu

**Affiliations:** ^1^Department of Psychiatry, Shanghai Mental Health Center, Shanghai Jiao Tong University School of Medicine, Shanghai, China; ^2^Shanghai Key Laboratory of Psychotic Disorders, Shanghai Mental Health Center, Shanghai Jiao Tong University School of Medicine, Shanghai, China

**Keywords:** major depressive disorder, cell adhesion molecule, protein microarray, diagnosis, plasma marker

## Abstract

**Objectives:** Major depressive disorder (MDD) is a serious mental disorder, and there is a great difficulty to diagnose and treat. Hitherto, relatively few studies have explored the correlation between the levels of plasma cell adhesion molecules and MDD.

**Methods:** Thirty outpatients with acute episodes of MDD in Shanghai Mental Health Center and 34 healthy volunteers from the community were recruited as subjects. Protein microarray technology was applied to compared the differences in plasma levels of 17 kinds of adhesion molecular proteins between the two groups. Meanwhile, the diagnostic value of different proteins in depression was discussed by using the receiver operating characteristic curve.

**Results:** The levels of Carcinoembryonic Antigen Related Cell Adhesion Molecule-1(CEACAM-1) and Neural Cell Adhesion Molecule (NrCAM) in MDD patients were significantly higher than those in healthy controls (*P* < 0.05). The area under ROC curve of CEACAM-1 combined with NrCAM was 0.723, with the sensitivity 0.800 and the specificity 0.676.

**Conclusion:** The plasma levels of CEACAM-1 and NrCAM were significantly up-regulated in MDD, and their combined application was of potential diagnostic value, deserving to expand the sample size for further verification.

## Introduction

Major depressive disorder (MDD) is a serious mental disorder with high prevalence rate, recurrence rate and disability rate (1, 2). According to the World Health Organization (WHO), it has become the leading contributor of disease burden that affects more than 264 million people worldwide (https://www.who.int/news-room/fact-sheets/detail/depression). However, the underlying molecular mechanism remains largely unknown. At present, clinical diagnosis of MDD mainly depends on the clinical manifestations that the patient presents as well as auxiliary application of rating instruments. Whereas, clinical symptoms may be dimensional and vary widely, leading to marked heterogeneity (3). In this context, the absence of objective biological detection methods makes the clinical diagnosis challenging. Exploring the biomarkers related to MDD for the diagnosis is an urgent need.

Cell Adhesion Molecules (CAMs) are transmembrane proteins located on the cell surface involved with the binding with other cells or with the extracellular matrix (4–6). CAMs mediate interactions between cells and their surroundings that are vital to processes controlling for cell proliferation, activation, migration, and survival (7). Based on their specific molecular structures, they are generally classified into four major CAM families: selectins, integrins, immunoglobulin (Ig)-like CAMs, and cadherins (8). CAMs play an important role in many vital physiological processes. Altered levels of CAMs can be found in diverse diseases. Recent advances have demonstrated that CAMs play a key role in several neurological and psychiatric diseases, such as Alzheimer's disease, MDD and schizophrenia (9–11). In addition, CAMs are involved in the inflammatory response, immunity and neuronal development in the brain (12). These functions have been proved to be closely related to MDD. Therefore, collective detection for these molecules would help us decipher their physiological functions.

Currently, the levels of most cytokines are detected through the use of the enzyme-linked immunosorbent assay (ELISA) (13). While this traditional method works well for a single protein, the overall procedure is time consuming and requires a relatively high volume of sample. With the advancement of microarray technology, this challenging task is effectively addressed (14). The Quantibody® array combines the advantages of the high detection sensitivity & specificity of ELISA and the high throughput of arrays. By arraying multiple cytokine specific capture antibodies onto a glass support, quantitative, multiplex detection of cytokines in one experiment is made possible.

Until now, the studies on the relationships between cell adhesion molecules levels in peripheral blood and MDD are scarce. In the present study, we examined the levels of 17 kinds of plasma cell adhesion molecules by protein microarray technology in depressed patients and healthy controls, in order to find out the possible biomarkers for identifying MDD.

## Materials and Methods

### Participants

Thirty patients with MDD were recruited from the outpatient clinic at the Shanghai Mental Health Center, Shanghai Jiao Tong University School of Medicine from June to September 2020. The inclusion criteria were as follows: (1) age between 18 and 60 years, gender unlimited; (2) all patients were assessed for meeting the “Diagnostic and Statistical Manual of Mental Disorders-fifth Edition” (DSM-5) for MDD; (3) first episode or recurrent, but none of the patients were medicated with antidepressants, electric convulsive therapy (ECT), modified ECT (MECT), repetitive transcranial magnetic stimulation (rTMS), or other physical therapy during the past 2 months; (4) the score of 17-item Hamilton Depression Rating Scale (17-HDRS) ≥17; (5) sufficient audiovisual ability and understanding of the Chinese language; (6) signed the informed consent form before participation. Patients with currently suffering from serious and physical diseases that may interfere with this research were excluded. The subjects with a history of any hypomanic or manic episodes were also excluded by the Mood Disorder Questionnaire (MDQ) and 32 items Hypomania Checklist (HCL-32). In addition, patients were excluded if they had serious suicidal ideation, attempt or behavior (e.g., score of item 3 of 17-HDRS ≥3). Pregnant or lactating patients were not eligible. A group of 34 sex-and age-matched healthy volunteers were recruited as controls by advertisement during the same period of MDD group recruitment. Participants were screened using the MINI-International Neuropsychiatric Interview (M.I.N.I.) and excluded if there were any history of psychiatric disorders. Similarly, any participant with severe somatic diseases was also excluded.

All recruited participants were of Chinese Han ethnicity and signed written informed consent. This study was approved by the Institutional Review Board of Shanghai Mental Health Center, Shanghai Jiao Tong University School of Medicine.

### Clinical Assessment

By using the self-designed questionnaire, we have obtained the general demographic information of the subjects and baseline clinical assessments data of the patients. Clinical assessments include number of depressive episodes, duration of disease, and previous treatment history. The 17-HDRS, Hamilton Anxiety Rating Scale (HAMA) and Inventory of Depressive Symptomatology Self Report (IDS-SR) were applied to assess the clinical symptoms of patients.

### Blood Sample Collection and Laboratory Test

Peripheral blood (5 ml) was collected from all participants in the morning and placed in EDTA anticoagulant vacutainer tubes. The plasma was obtained by centrifuging (3,000 rpm, 4°C, 15 min) and then stored in the −80°C refrigerator. Repeated freezing and thawing were avoided. The levels of cell adhesion molecule proteins in the plasma were detected using RayBiotech biotin-labeled antibody chip (Guangzhou Ruiboao Biotechnology Co., Ltd, QAH-CAM-1 kit). All operation steps were carried out in accordance with the kit instructions. The plasma was diluted in different proportions according to different cell adhesion molecule concentrations. After the chip was completely dried, it was sealed and incubated, followed by fluorescence detection. The signal was scanned by a laser scanner InnoScan 300 with the scanning parameters of Wavelength 532 nm and Resolution 10 μm. QAH-CAM-1 data analysis software was used for data analysis.

### Statistical Analyses

The demographic and clinical characteristics that met the normal distribution were described in the form of mean ± standard deviation (S.D.), otherwise were expressed as median (lower quartile, upper quartile). The cell adhesion molecule expression levels of all the subjects were normalized using the average value. Antibody with detectable signal intensities in <20% of all analyzed samples were excluded. The average value filled the remaining missing values, and the average value was standardized. For comparisons between groups, continuous variables were compared using independent samples *t*-test, and categorical variables were compared using chi-square test. Partial correlation analysis was used to analyze correlation between clinical symptoms and the levels of proteins. In this study, we performed logistic regression models with two differential CAMs as the independent variable, and whether depression as the dependent variable. The predictive value of the model was evaluated by receiver operating characteristic curve (ROC). All statistical analyses were performed using R statistical package (R version 4.0.2). Differences were considered statistically significant if the *P*-value (two-tailed) was < 0.05.

## Results

### Demographic and Clinical Characteristics

The demographic and clinical characteristics of all subjects are presented in Table 1. No significant difference in gender and age were found between the patient and control groups (*P* > 0.05). There were 23 first-onset patients and 7 recurrent patients of all 30 MDD patients. The median duration of current episode was 3.50 months (range, 1.00, 13.00 months) and total duration was 6.00 months (range, 2.00, 30.00 months).

**Table 1:** Demographic and clinical characteristics of MDD and HC subjects.

**Variables**	**MDD (*n* = 30)**	**HC (*n* = 34)**	****χ**^2^/t**	***p*-value**
Gender (female, %)	25, 83%	22, 65%	χ^2^ = 1.960^a^	0.162
Age (years)	27.13 ± 6.85	29.09 ± 8.08	*t* = 1.048^b^	1.299
Duration of this episode (month)	3.50 (1.00, 13.00)	N.A.		
Total duration of disorder (month)	6.00 (2.00, 30.00)	N.A.		
17-HDRS score	25.40 ± 4.33	N.A.		
HAMA score	22.23 ± 7.05	N.A.		
IDS-SR score	41.97 ± 12.79	N.A.		

*MDD, major depressive disorder; HC, healthy controls; N.A., not applicable*.

a *Chi-square test*;

b *Independent samples t-test*.

### The Expression Levels of Cell Adhesion Molecules in Plasma

As showed in Table 2, the comparison of cell adhesion molecules levels in plasma between MDD patients and healthy controls demonstrated statistically significant (*P* < 0.05) for carcinoembryonic antigen-related cell adhesion molecules (CEACAM-1) and neural cell adhesion molecules (NrCAM), but not for other CAMs (*P* > 0.05). Compared to the control group, plasma levels of CEACAM-1, and NrCAM in MDD were significantly elevated. No significant correlations between the level of CEACAM-1 in plasma and the total score of 17-HDRS (*r* = 0.095), HAMA (*r* = −0.129), and IDS-SR (*r* = 0.030) (*P* > 0.05) were shown. There were no statistically significant correlations between the level of NrCAM in plasma and the total score of 17-HDRS (*r* = −0.157), HAMA (*r* = 0.158) and IDS-SR (*r* = 0.056), either (*P* > 0.05).

**Table 2:** The levels of CAMs in plasma from MDD patients compared to HCs.

**CAMs (pg/ml)**	**MDD (*n* = 30)**	**HC (*n* = 34)**	***t***	***p*-value**
ALCAM	0.20, 0.12	0.18, 0.11	−0.887	0.379
BCAM	4.11, 4.90	2.76, 3.04	−1.305	0.198
CEACAM-1	3.18, 1.15	2.54, 1.19	−2.189	**0.032**
E-Cadherin	2.24, 4.71	1.41, 5.61	−0.642	0.523
EpCAM	0.71, 0.60	0.59, 0.41	−0.909	0.368
E-Selectin	25.14, 50.02	12.31, 32.82	−1.162	0.251
ICAM-1	21.63, 5.88	24.13, 4.62	1.874	0.066
ICAM-2	101.34, 33.28	103.51, 33.61	0.260	0.796
ICAM-3	1.75, 3.55	0.88, 1.49	−1.251	0.219
L-Selectin	323.33, 55.85	338.70, 44.28	1.209	0.232
NCAM-1	83.20, 25.88	84.56, 18.30	0.240	0.811
NrCAM	1.92, 1.830	0.99, 0.797	−2.582	**0.014**
P-Cadherin	37.46, 9.51	34.93, 6.67	−1.218	0.229
PECAM-1	11.17, 13.25	7.40, 9.17	−1.308	0.197
P-selectin	18.75, 38.83	9.66, 25.01	−1.097	0.278
VCAM-1	345.19, 79.82	362.01, 54.57	0.971	0.336
VE-Cadherin	18.67, 11.68	13.45, 8.97	−1.988	0.052

*ALCAM, Activated leukocyte cell adhesion molecule; BCAM, Basal cell adhesion molecule; E-Cadherin, Epithelia-cadherin; EpCAM, Eithelial cellular adhesion molecule; ICAM-1, Intercellular adhesion molecule-1; ICAM-2, Intercellular adhesion molecule-2; ICAM-3, Intercellular adhesion molecule-3; NCAM-1, Neural cell adhesion molecule-1; P-Cadherin, Placental Cadherin; PECAM-1, Platelet endothelial cell adhesion molecule-1; VCAM-1, Vascular cell adhesion molecule-1; VE-Cadherin, Vascular endothelial cadherin. Bold value indicates statistically significant (P < 0.05)*.

### Logistic Regression Model Development

Two kinds of CAMs with significant differences between the two groups were screened out by *t*-test. Thus, the association between two differential CAMs and depression was assessed using multivariate logistic regression model. Regression coefficients and Odds Ratios (OR) are provided in Table 3.

**Table 3:** Coefficients from the logistic regression model.

**Variables**	**Estimate**	**Std.Error**	**95% CI for Estimate**	**Pr(>|z|)**	**OR**	**95% CI for OR**
(Intercept)	−1.815	0.753	−3.401 ~−0.414	0.016	0.163	0.033 ~ 0.661
NrCAM	0.59	0.302	0.080 ~ 1.269	0.051	1.803	1.083 ~ 3.558
CEACAM-1	0.318	0.247	−0.152 ~ 0.829	0.198	1.374	0.859 ~ 2.291

*OR, odds ratios; CI, confidence interval*.

### Joint Validation of Two Differential Proteins

**Figure 1:**
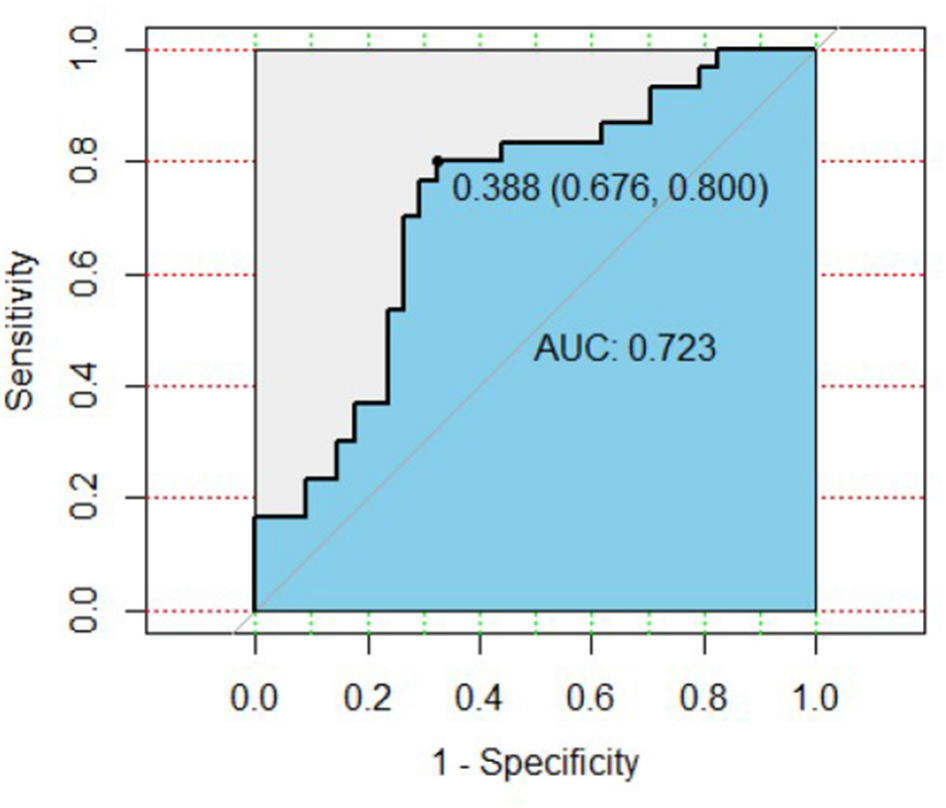
ROC curve of plasma CEACAM-1 combined with NrCAM for the diagnosis of MDD. AUC, area under the ROC curve; ROC, receiver operating characteristic.

To assess the ability of two different proteins to predict MDD, we applied ROC analysis. The ROC curve of CEACAM-1 combined with NrCAM to distinguish MDD patients from healthy controls is shown in Figure 1. It showed an area under the ROC curve (AUC) was 0.723. The model, with a cutoff of 0.388, revealed a sensitivity and specificity of 0.800 and 0.676, respectively.

## Discussion

MDD is one of the most prevalent mental diseases that leads to individual suffering, as well as major economic and societal burdens (15). Considering the high heterogeneity and complex pathogenesis, identification and diagnosis of MDD remain a huge global challenge. Up to date, no objective detecting method has been fully certificated. Thus, exploring potential biomarkers might be helpful for clinicians to make an objective and accurate diagnosis for MDD. In this study, we analyzed the plasma levels of 17 cell adhesion molecules in 30 MDD patients and 34 healthy controls. Levels of CAMs were measured utilizing antibody microarray technology instead of one in a traditional ELISA. In contrast to ELISA, innovations in microarray technology allowed us to simultaneously quantify and analyze multiple target biomarkers by this throughout technique. The microarray technology has certain advantages in sensitivity and detection the lowest concentrations of cytokines, which has been confirmed by previous studies (16, 17). The results demonstrated that the plasma CEACAM-1 and NrCAM levels of MDD patients were significantly higher than that of healthy controls. Notably, AUC value was 0.723 which indicated that plasma CEACAM-1 combined with NrCAM might have some predictive value for diagnosis of MDD. The model, with a cutoff of 0.388, presented the sensitivity and specificity of 0.800 and 0.676, respectively. To our knowledge, this is the first preliminary exploration of cell adhesion molecules levels in plasma from MDD patients by high throughout protein microarray technology.

CAMs are specialized proteins usually expressed on the cell surface, which play a key role in synaptic plasticity, synaptic function and neural circuit remodeling (18, 19). Recently, several studies have suggested prominent role of CAMs in the pathophysiology of mental disorders (20). Nevertheless, little was found in the literature on the associations between CAMs and MDD. Previous studies have shown that NrCAM imbalance might be involved in the occurrence of MDD and NrCAM served as one of risk factors for mental illnesses (10, 21, 22). NrCAM, alternatively called CD56, is a member of the Ig-superfamily (23). NCAM was found in nearly all tissues, but the highest expression was found in central and peripheral nerve tissues (24). Animal studies have suggested that NrCAM-deficient mice exhibited a depression-like behavior and altered hippocampal plasticity (21). In addition, chronically stressed rats displayed reduced NrCAM mRNA and protein levels in the hippocampus (25, 26). These results were validated in some clinical studies. One clinical investigation has detected increased levels of NrCAM in the cerebrospinal fluid (CSF) of patients with unipolar depressive disorder (27). However, this was not in line with the findings from Hidese et al. (20), which found NCAM was down-regulated in CSF of MDD compared with heathy controls (20). The reasons for this discrepancy could be due to sample size, disease course, use of antidepressants, and protein determination techniques. Some studies have shown that the function of NrCAM depends on its glycosylation, in particular polysalification, known as polysialic acid (PSA) (28). There is growing evidence which support the importance of polysialylated NCAM (PSA-NCAM) in MDD (19). It has been reported that the expression of PSA-NrCAM was reduced in MDD patients and in animal models of depression, while antidepressant treatment increased expression of PSA-NrCAM (19, 29, 30). Not only that, NrCAM functions at the neural plasticity, involving the theories of monoamine and neurotrophin in depression (19). At present, there are few studies on the relationship between plasma NrCAM and MDD, mainly focusing on CSF. The reason is that CSF is the optimal biological material for examination of molecular status, and has been reported to well reflect the state of the central nervous system (31–33). Many active substances, such as vascular endothelial growth factor (VEGF), Interleukin (IL)-1, IL-6, and reactive oxygen species (ROS), are closely related to the destruction of the integrity of the blood-brain barrier (BBB), and these substances are also important members of the pathogenesis of MDD (34). Thus, patients with MDD may have an increase in the permeability of the BBB. In this study, we hypothesized that the changes of NrCAM in the CSF of patients with MDD might be reflected in the peripheral blood of patients. Our study found that higher plasma NrCAM levels in patients with MDD, compared to healthy controls.

Interestingly, we found that CEACAM-1 is also upregulated in plasma of MDD compared with healthy people. CEACAM-1, also known as CD66a, is a transmembrane glycoprotein, and belongs to the carcinoembryonic antigen family (35). CEACAM-1 has been found to be up-regulated in some cancers (36). Recent studies have found that CEACAM-1 mediates inflammatory responses as an inflammatory factor and plays an interactive regulatory role with inflammatory mediators. Literature have reported that CEACAM-1 plays a regulatory role on IL-6, and CEACAM-1 knockout mice shows a higher level of IL-6 (37). On top of this, the phosphatidylinositol-3-kinase/Akt (PI3K/Akt) pathway can induce the upregulation of CEACAM-1 stimulated by γ-IFN, while CEACAM-1 can also promote the activation of PI3K/Akt (38). In recent years, MDD is considered as an inflammatory disease. Results from numerous studies have confirmed that elevated levels of pro-inflammatory cytokines in MDD patients, such as IL−6 and γ-interferon (IFN) (39). Although the role of CEACAM-1 in MDD remains unclear, our results suggest that it may be a potential biomarker.

The present study has certain limitations that should be taken into account. While we found differences in NrCAM and CEACAM-1 between MDD patients and healthy controls, the changes were not associated with depression severity. It was due to relatively small size, which needs further clarification in larger cohorts in order to better evaluate the correlation between cell adhesion molecules and MDD. Also, this is a preliminary study with cross-sectional design which is hard to reflect longitudinal alteration of cell adhesion molecules before and following treatment. Therefore, we could not draw the conclusion that CEACAM-1 and NrCAM are state or strait biomarkers for MDD.

In conclusion, the results demonstrate that plasma of CEACAM-1 and NrCAM levels were significantly up-regulated in medication-naïve MDD patient, suggesting that CAMs might play a role in pathophysiological mechanisms of MDD. Combined application of CEACAM-1 and NrCAM might be of potential diagnostic value. Our findings deserve further validation in big sample size and well-designed studies in future.

## Data Availability Statement

The original contributions presented in the study are included in the article/supplementary material, further inquiries can be directed to the corresponding author/s.

## Ethics Statement

The studies involving human participants were reviewed and approved by Ethics Committee of Shanghai Mental Health Center. The patients/participants provided their written informed consent to participate in this study.

## Author Contributions

WL and YZ performed the statistical analyses and wrote the manuscript. FZ and MZ completed all of the data entry. TZ and QG managed the literature searches and analyses. HX, CL, HC, XW, YH, and GL were responsible for the diagnosis and clinical assessment of the participants. ZL and CZ provided assistance for laboratory work. KJ and XL offered many constructive opinions on this study and provided a critical revision for the manuscript. All authors contributed to and approved the final manuscript.

## Conflict of Interest

The authors declare that the research was conducted in the absence of any commercial or financial relationships that could be construed as a potential conflict of interest.
